# Phase I study targeting newly diagnosed grade 4 astrocytoma with bispecific antibody armed T cells (EGFR BATs) in combination with radiation and temozolomide

**DOI:** 10.1007/s11060-024-04564-y

**Published:** 2024-01-23

**Authors:** Camilo E. Fadul, Archana Thakur, Jungeun Kim, Jessica Kassay-McAllister, Dana Schalk, M. Beatriz Lopes, Joseph Donahue, Benjamin Purow, Patrick Dillon, Tri Le, David Schiff, Qin Liu, Lawrence G. Lum

**Affiliations:** 1 Department of Neurology, Division of Neuro-Oncology, University of Virginia Health System, P.O. Box 800394, Charlottesville, VA 22908 USA; 2 Bone Marrow Transplant Program, Division Hematology/Oncology, Department of Medicine, University of Virginia Cancer Center, Charlottesville, VA USA; 3 Office of Clinical Research, School of Medicine, University of Virginia Cancer Center, Charlottesville, VA USA; 4 Department of Pathology, Divisions of Neuropathology, University of Virginia Health System, Charlottesville, VA USA; 5 Department of Radiology and Medical Imaging, Division of Neuroradiology, University of Virginia Health System, Charlottesville, VA USA; 6 Division Hematology/Oncology, Department of Medicine, University of Virginia Cancer Center, Charlottesville, VA USA; 7 Biostatistics Unit, Molecular & Cellular Oncogenesis Program, The Wistar Institute, Philadelphia, PA USA

**Keywords:** Glioblastoma, Immune therapy, EGFR, Bispecific antibodies, Phase I

## Abstract

**Purpose:**

The purpose of this study was to determine the safety, feasibility, and immunologic responses of treating grade 4 astrocytomas with multiple infusions of anti-CD3 x anti-EGFR bispecific antibody (EGFRBi) armed T cells (EGFR BATs) in combination with radiation and chemotherapy.

**Methods:**

This phase I study used a 3 + 3 dose escalation design to test the safety and feasibility of intravenously infused EGFR BATs in combination with radiation and temozolomide (TMZ) in patients with newly diagnosed grade 4 astrocytomas (AG4). After finding the feasible dose, an expansion cohort with unmethylated *O*^*6*^*-methylguanine-DNA methyltransferase* (MGMT) tumors received weekly EGFR BATs without TMZ.

**Results:**

The highest feasible dose was 80 × 10^9^ EGFR BATs without dose-limiting toxicities (DLTs) in seven patients. We could not escalate the dose because of the limited T-cell expansion. There were no DLTs in the additional cohort of three patients with unmethylated MGMT tumors who received eight weekly infusions of EGFR BATs without TMZ. EGFR BATs infusions induced increases in glioma specific anti-tumor cytotoxicity by peripheral blood mononuclear cells (*p* < 0.03) and NK cell activity (*p* < 0.002) ex vivo, and increased serum concentrations of IFN-γ (*p* < 0.03), IL-2 (*p* < 0.007), and GM-CSF (*p* < 0.009).

**Conclusion:**

Targeting AG4 with EGFR BATs at the maximum feasible dose of 80 × 10^9^, with or without TMZ was safe and induced significant anti-tumor-specific immune responses. These results support further clinical trials to examine the efficacy of this adoptive cell therapy in patients with MGMT-unmethylated GBM.

**ClinicalTrials.gov Identifier**: NCT03344250

**Supplementary Information:**

The online version contains supplementary material available at 10.1007/s11060-024-04564-y.

## Introduction

Glioblastoma (GBM) is the most frequent type of primary malignant brain tumor, with an estimated 13,000 new cases in the US in 2023 [1]. Despite standard surgical resection followed by radiation therapy (RT) and temozolomide (TMZ), the median overall survival (OS) for patients with unmethylated *O*^*6*^*-methylguanine-DNA methyltransferase* (MGMT) is 12.6 months and with methylated MGMT is 23.4 months, respectively [2, 3]. Although immunotherapy has improved the outcomes associated with many types of cancer, most clinical trials examining different approaches for GBM have been disappointing [4–8]. The unique features of the immune tumor microenvironment (TME) in the central nervous system (CNS) system and the overwhelming immunosuppression associated with GBM make therapeutic manipulation of the immune system a formidable challenge. To be effective, immunotherapy-targeting GBM needs to overcome systemic and local suppression, cross the blood-brain barrier (BBB), and induce a therapeutic anti-tumor effect.

Glioblastoma cells overexpress epidermal growth factor receptor (EGFR) to varying degrees and have been a target for previous immunotherapies, though with unsatisfactory results [9–11]. Adoptive immune cell therapy has the potential to overcome the problem of delivery and adaptive mechanisms of resistance thought to be, at least in part, responsible for the failure of therapeutically targeting EGFR in GBM [12]. Anti-CD3 monoclonal antibody activated T cells (ATC) armed with anti-CD3 x anti-EGFR bispecific antibody (EGFR BiAb) target T cells to tumor-associated antigen (TAA) on solid tumors in a non-MHC restricted manner [13]. Our preclinical studies showed that EGFRBi-armed T cells (EGFR BATs) lyse both established and patient-derived GBM cell lines, and induce multiple rounds of proliferation, endogenous tumor-specific cytotoxicity, and Th_1_ cytokine release without affecting T cell viability. The range of antitumor and immunostimulatory complementary functions may overcome the limitations of other types of cancer immunotherapy [11, 14].

This phase I study in patients with astrocytoma grade 4 (AG4) [15] examines whether infusions of EGFR BATs in combination with standard radiation and chemotherapy are safe and feasible and if the therapy induces systemic anti-tumor immunity. This study shows that a total of 80 × 10^9^ EGFR BATs is safe without cytokine release syndrome (CRS) and induces anti-tumor immunity.

## Methods

### Study design

**Fig. 1:**
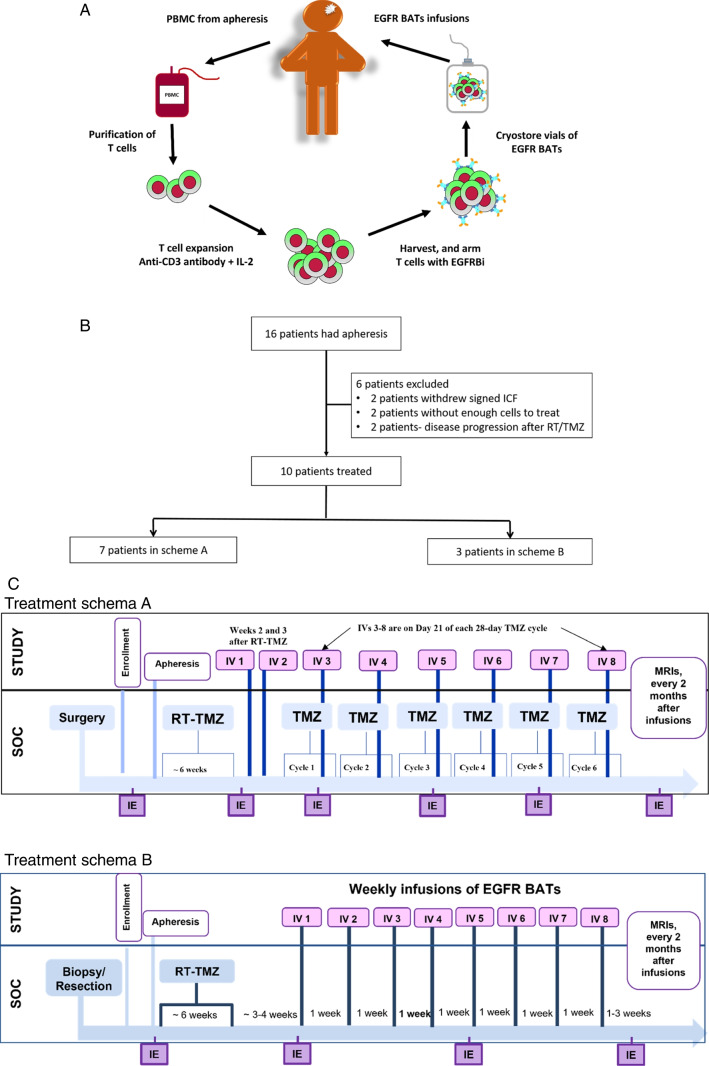
**A** EGFR BATs manufacturing schema from the collection of apheresis product, T cell expansion, loading ATC with BiAb, cryopreservation, and infusion of cell product into patients. **B** Flow diagram of patients enrolled in the study. **C** Treatment schemas. SOC: standard of care; IE: immune evaluations;  IV: intravenous, RT: radiation therapy; TMZ: temozolomide

This study was a single-center phase I dose-escalation trial using a standard 3 + 3 design to determine the maximum tolerated and feasible dose (MTFD) of EGFR BATs in combination with standard of care (SOC) in patients with newly diagnosed GBM [16]. Patients 18 years or older with histologically GBM, according to the 2016 World Health Organization classification of tumors of the CNS, were eligible for screening. Molecular confirmation of MGMT status was required for all patients. Inclusion criteria included a Karnofsky Performance Scale (KPS) score of 60 or above and required baseline laboratory data. After the planned upper two dose levels were not feasible due to limited cell yields, the protocol was amended to include another cohort with unmethylated MGMT to determine if eight weekly IV doses of ~ 8 × 10^9^ EGFR BATs/infusion without adjuvant TMZ (Fig. 1C) was safe. Patients in the first cohort required maximal safe tumor surgical resection and a brain MRI within 72 h after surgery before consent for EGFR BAT therapy. For patients in the second cohort, a biopsy was acceptable if surgical debulking was not indicated. Exclusion criteria included pregnancy, ongoing immunosuppressive therapy except for corticosteroids, and active systemic bacterial, viral, or fungal infection. We excluded patients with alpha-1,3 galactose IgE (alpha-gal) test results outside of the reference range, which could indicate hypersensitivity to the cetuximab component of the BiAb [17]. The first dose level (10 × 10^9^ EGFR BATs/infusion) was given with the possibility of escalating to dose levels two (15 × 10^9^ EGFR BATs/infusion) and three (20 × 10^9^ EGFR BATs/infusion) for subsequent cycles. Patients had cells collected by apheresis before initiating standard concurrent RT and TMZ. Patients received the first and second infusions of EGFR BATs on days 14 and 21 after finishing concurrent RT and TMZ and then received an infusion on day 21 of the first six cycles of TMZ. Patients had a brain MR approximately one week before the first EGFR BATs treatment. If patients were symptomatic from edema requiring a dose ≥ 4 mg of dexamethasone per day, they were withdrawn from the study. Patients who received at least one infusion of EGFR BATs were assessed for toxicity and survival, only patients who received at least two infusions were considered evaluable for dose escalation.

### Trial oversight

 This protocol was approved by the University of Virginia (UVA) Institutional Review Board (IRB-HSR: 20105) and the FDA, BB-IND #13091. The study was monitored by the UVA Cancer Center Data and Safety Monitoring Committee.

### Endpoints

 The primary endpoints were safety and feasibility. We determined toxicity according to the NCI Common Toxicity Criteria for Adverse Events (CTCAE, 4.03). Dose-limiting toxicities were identified during the EGFR BATs infusions and up to 7 days after each infusion and for 30 days after the last infusion. We defined the feasible dose for an eligible participant as achieving at least 80% of the planned dose.

Secondary endpoints included immune responses as measured by cellular phenotype, interferon-γ (IFN-γ) EliSpots (a surrogate marker of anti-GBM cytotoxicity) of fresh peripheral blood mononuclear cells (PBMC) directed at GBM cell lines, and serum cytokine patterns at various time points shown in the treatment schema (Fig. 1C) as IE (immune evaluation time points) [18].

We performed a preliminary assessment of PFS and OS, defined from the time patients had the apheresis. We used the Response Assessment in Neuro-oncology (RANO) criteria modified for immunotherapy (iRANO) but follow-up imaging was done within 2 instead of 3 months after initial radiographic progression if there was no new or substantially worsened neurological deficits that were not due to comorbid events or concurrent medication [19].

### Activation, expansion, and arming of T cells

The EGFRBi was produced and the T cells in PBMC were activated and expanded as previously described [20]. Activated T cells were harvested, armed with EGFRBi, and then washed to remove unbound EGFRBi and cryopreserved in eight equal aliquots until they were thawed for infusion (Fig. 1A). Based on the viability, phenotype, and functional activity, product stability was 6 months from the time of cryopreservation of the product.

### Quality assurance of EGFRBi armed ATC cell product and release criteria

The final EGFR BATs product was released for administration after testing for sterility, mycoplasma, and endotoxin [21]. At the time of harvesting, several cryovials of EGFR BATs were cryopreserved for QC testing. Release criteria for armed products for clinical use included negative results for bacterial cultures, fungal cultures, mycoplasma, and endotoxin assays (endotoxin < 2.5 EU/mL). The release criteria for functional activity of the EGFR BATs product included 70% viability, and ≥ 10% cytotoxicity directed at EGFR + target cells. The proportion of CD3 + , CD19, CD20, CD16, and CD56 cells was recorded.

### Statistical analysis

The primary objective of this dose escalation study was to determine the MTFD of EGFR BATs at dose levels 10, 15, and 20 × 10^9^ per infusion respectively. Dose level one was eight infusions of 10 × 10^9^ of EGFR BATs. If two or more out of three, or two or more out of six patients had DLTs at dose level one, the trial would be stopped. The same rule applied for dose levels two and three. For immune responses, we calculated point estimates and generated 95% confidence intervals. Estimates of PFS and OS curves were plotted generating Kaplan-Meier curves using GraphPad Prism version 9.0.0 for Windows (GraphPad Software, San Diego, California USA, www.graphpad.com). Immune responses were compared at the highest response post BATs infusions with pre-infusion baseline IFN-γ EliSpots. Differences between serum cytokines/chemokines and CTL activity directed at U87 and K562 cell lines were analyzed using the Wilcoxon signed-rank test.

## Results

### Patients

The study started in October 2017 and accrual to the dose-escalation cohort was halted in January 2020 because the upper two dose levels were limited by cell yields from a single apheresis. The protocol was amended to determine the safety of eight weekly doses of EGFR BATs without adjuvant TMZ in patients with unmethylated MGMT. The study was offered to 21 patients with newly diagnosed AG4 who signed informed consent for eligibility. Four patients (20%) were not eligible due to elevated Alpha-Gal and one patient (5%) was ineligible due to a positive-Hepatitis B test. Of the 16 patients who had apheresis, six patients were not treated because of an insufficient yield after expansion (two patients), functional status declined after RT/TMZ (two patients), or withdrawn consent (two patients) (Fig. 1C). Fifty percent of the tumors had hypermethylated MGMT. Two tumors had R132H mutant isocitrate dehydrogenase (IDH) and the others had wildtype IDH (Table 1). According to the 2021 WHO classification, diffuse infiltrating gliomas with grade 4 gliomas histologic characteristics and IDH mutation are designated as astrocytoma, IDH-mutant, grade 4 [15]. All patients had astrocytomas grade 4 (AG4), eight IDH wildtype (GBM), and two mutated (AG4).

**Table 1:** Patient and tumor characteristics, treatment, and outcomes

Patient	Age	Sex	IDH	KPS	MGMT	Surgery	No. of Infusions	EGFR BATs	Total Dose (× 10^9^)	Steroid dose	PFS (months)	OS (months)
								dose (× 10^9^)	Infused	mg/day		
^A^GBM6	70	F	WT	60	Met	GTR	8	10	80	1	26.2	41.5
^A^GBM7	28	M	MUT	90	Met	Partial	2	10	20	0	22.1	39.9
^A^GBM9	42	F	WT	80	U	GTR	8	10	80	1	16	31.0
^A^GBM11	69	M	WT	80	Met	Partial	8	7	56	0	18.2	26.4
^A^GBM12	59	M	WT	70	Met	Partial	2	15	30	1	31.3	46.3
^A^GBM14	22	F	MUT	100	Met	Partial	8	15	103	0	29.5	43.0
^A^GBM15	55	F	WT	90	U	GTR	8	15	104	0	9.7	16.6
^B^GBM17	73	M	WT	70	U	Biopsy	8	10	80	1	8.5	15.8
^B^GBM21	64	F	WT	70	U	Biopsy	8	10	80	4	4.8	6.0
^B^GBM23	48	F	WT	80	U	Partial	8	10	80	2	6.7	13.2

*A* these patients received treatment schema A with adjuvant TMZ, *B* these patients received treatment schema A with adjuvant TMZ, *IDH* isocitrate dehydrogenase, *WT* wild-type, *MUT* mutated, *MGMT O*^*6*^*-methylguanine-DNA methyltransferase*, *Met* methylated, *U* unmethylated, *GTR* gross total resection; steroid dose at the time of first infusion, *PFS* progression-free survival, *OS* overall survival

### Toxicity

For the ten patients who received BATs infusions ± adjuvant TMZ, we found no DLTs or persistent grade 3 or 4 toxicities (Table 2). No AEs led to treatment discontinuation except for one patient who had intratumoral bleeding after the second infusion. However, the relationship to EGFR BATs was unclear because the patient had intratumoral bleeding before the study infusions. Another patient was withdrawn after two infusions because of non-compliance. Eight patients completed the eight planned infusions. There were 68 infusions with the most frequent Grade 3 or above side effects related to the infusion being headache, hypertension, and hypotension, all resolving within 24–48 h of infusions. Otherwise, the most common AEs were lymphopenia, thrombocytopenia, and fatigue (Table 2). Possibly or definitely treatment-related AEs included seizures (3) and intracranial hemorrhage (1). Table S1 shows the AEs that occurred in ≤ 10% of infusions.

**Table 2:** Adverse events associated with 68 infusions of EGFR BATs (> 10% of infusions)

Adverse event	Grade 1–2	Grade 3	Grade 4	Total No of events (%)
Lymphopenia	18	15	6	39 (57)
Headache	20	1	0	21 (31)
Thrombocytopenia	15	2	1	18 (26)
Hypertension	12	5	0	17 (25)
Hypotension	14	2	0	16 (24)
Fatigue	14	0	0	14 (21)
Chills	13	0	0	13 (19)
Nausea	9	0	0	9 (13)
Fever	8	0	0	8 (12)

### Feasibility

Two of four patients assigned to the second total dose level of 120 × 10^9^ were not treated at that dose; in one case, ATC expanded but did not survive cryopreservation and in the other, the ATC quantity was insufficient upon thaw to treat the lowest dose level. Therefore, the recommended phase II dose was 80 × 10^9^ EGFR BATs. The additional cohort of three patients with unmethylated-MGMT GBM were treated with eight weekly infusions of 10 × 10^9^ EGFR BATS instead of adjuvant TMZ without DLTs (Fig. 1C).

### Clinical outcomes

 All patients had progression of the disease and died, with a median PFS of 17.2 months and a median OS of 28.8 months **(**Figs. S1 and S2).

### Characteristics of cell therapy product

 The mean and range of each immune cell subset, CD4/CD8 ratios in ATC, and specific cytotoxicity of the product are shown in Table S2.

### Post therapy immune monitoring

### Enhanced CTL and natural killer (NK) activity in PBMC after infusions of EGFR BATs

**Fig. 2:**
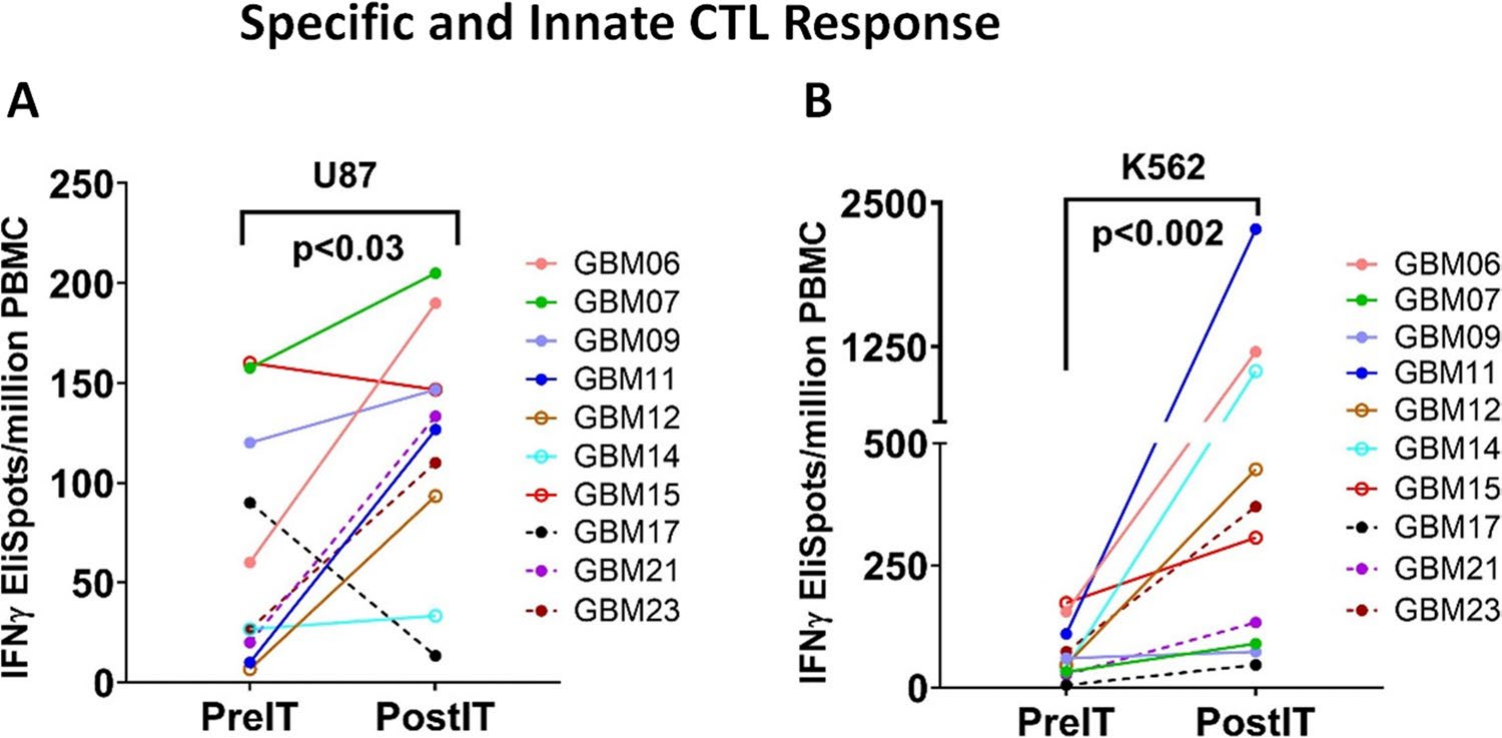
IFN-γ EliSpots PostIT compared to PreIT baseline responses against (**A**) U-87 cell line(*p* < 0.03). **B** NK target K562 cells (*p* < 0.002). Three out of 10 patients who received weekly infusions of EGFR BATs are shown by the dashed line or open shapes and seven patients who received monthly infusions of EGFR BATs are shown by solid line. Each patient is the same color-coded at pre- and postIT time points

 To assess the development of endogenous anti-tumor activity, fresh PBMC were tested for cytotoxic T lymphocyte (CTL) activity by measuring the IFN-γ ELISpots upon stimulation with GBM cell line U87 and NK cell-specific target K562 preimmunotherapy (PreIT) and post-immunotherapy time points (PostIT). Because the timing of the immune response varied, the highest single PostIT time point CTL response was compared with the PreIT (baseline) time point. There were significantly increased tumor-specific IFN-γ responses (*p* < 0.03) against U87 cells in the PostIT sample at the time point of peak immune response compared with PreIT samples (Fig. 2A). Likewise, significantly enhanced IFN-γ activity (*p* < 0.002) was seen at PostIT against NK sensitive target K562 cells compared to PreIT PBMC (Fig. 2B).

#### Enhanced Th_1_ cytokine and chemokine responses after EGFR BATs infusions

**Fig. 3:**
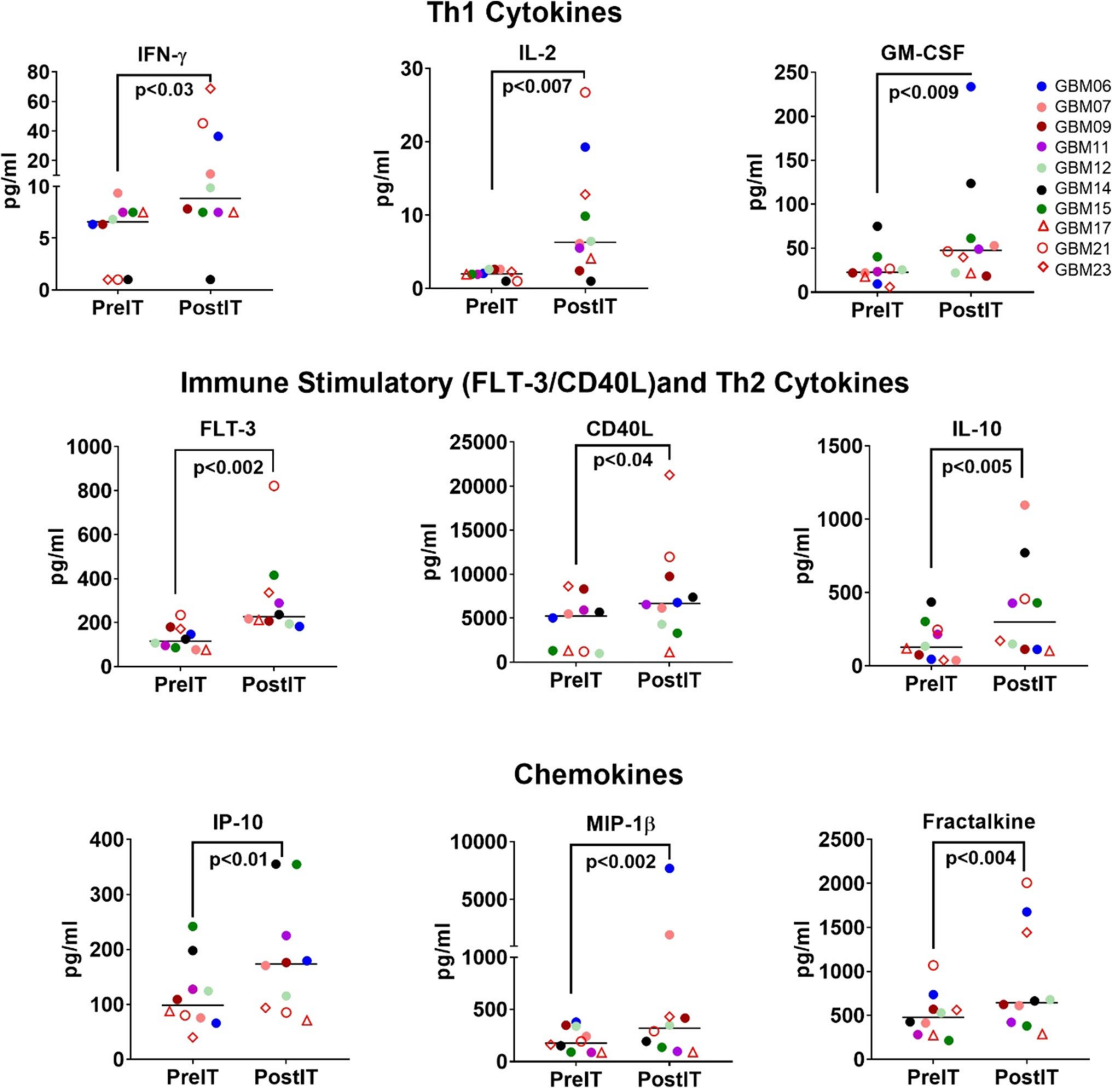
The upper panel shows PreIT and Post IT serum samples after EGFR BATs infusions for the Th_1_ cytokine IFN-γ, IL-2, and GM-CSF. The middle panel shows PreIT and Post IT levels for the Th_2_ cytokines IL-10, Flt3L, and CD40L. The lower panel shows levels of chemokines IP-10, MIP-1β, and fractalkine PreIT and PostIT. Open shapes show three out of ten patients who received weekly infusions of EGFR BATs and seven patients who received monthly infusions of EGFR BATs are shown by solid dots. Each patient is the same color-coded at Pre- and PostIT time points

Serum samples obtained at baseline (PreIT) and after EGFR BATs infusions (PostIT) were tested with a panel of 27 cytokines and chemokines. Table S3 shows the fold change for selected cytokines or chemokines at PreIT and a single time point PostIT that showed the highest cytokines or chemokines response. Cytokines and chemokines that showed significant change after EGFR BATs infusion compared to pre-infusion (preIT) include Th_1_ and Th_2_ cytokines (IL-2, IFN-γ, and GM-CSF, IL-10), chemokines (IP-10, MIP-1β, and Fractalkine) and ligands for co-stimulatory (CD40L) and growth and differentiation receptors (Flt3L) are shown in Fig. 3. The Th_1_ cytokines IFN-γ (*p* < 0.03), IL-2 (*p* < 0.007), and GM-CSF (*p* < 0.009) (Fig. 3, upper panel), and one Th_2_ cytokine, IL-10, increased significantly after EGFR BATs. Both Flt3L (*p* < 0.002) and CD40L (*p* < 0.04), which have each been shown to have immune-stimulatory and anti-tumor effects, increased significantly at PostIT compared to PreIT (Fig. 3, middle panel) [20]. The IFN-induced chemokine IP-10 increased significantly (*p* < 0.01) at PostIT, along with T cell recruiting chemokines MIP-1β (*p* < 0.002) and Fractalkine (*p* < 0.004), compared to PreIT concentrations (Fig. 3, lower panel).

## Discussion

To circumvent and overcome the limitations of monoclonal antibody therapy targeting EGFR alone in patients with GBM, we designed this phase I study based on our preclinical data that showed that the arming of ATC with EGFR BiAb exerts potent in vitro anti-tumor activity [11]. The combination of intravenous administration of EGFR BATs after RT/TMZ in patients with newly diagnosed AG4 was safe using two different regimens: every 4 weeks in combination with TMZ and weekly as the only adjuvant treatment after standard RT-TMZ. The starting total dose of 80 × 10^9^ EGFR BATs given in eight divided doses was selected based on previous trials using EGFR BATs to start the dose escalation with the highest safe dose possible to maximize the trafficking of BATs across the BBB [22]. Expanding cells from single apheresis was not sufficient to attain the second dose level. The safe and feasible phase II recommended dose of 80 × 10^9^ cells in eight divided infusions triggered adaptive and endogenous anti-tumor cellular immune responses in PBMC of patients with AG4 with the potential to translate into therapeutic benefit.

A concern with this immunotherapy strategy is that it might cause a CRS similar to that seen after chimeric antigen receptor (CAR)T cell infusions, but we found no DLTs, and most of the observed adverse events resolved within 24–48 h after the infusion. This side effect profile was similar to our previous experience using BATs in patients with other types of cancer [21–24]. A patient developed a symptomatic intratumoral hemorrhage, but the tumor had bled before immunotherapy. The safety profile of EGFR BATs in patients with GBM will be confirmed in a larger phase II cohort.

Patients with GBM are immunosuppressed with baseline lymphopenia, have T-cell dysfunction, and exhibit higher proportions of Tregs in peripheral blood and the tumor microenvironment [25, 26]. Although the T cells in the apheresis product before RT/TMZ did not contain Tregs, T cell expansion was insufficient to fully explore dose level 2. However, EGFR BATs products from patients with AG4 and healthy controls showed comparable in vitro cytotoxicity (data not shown). Despite impaired ex vivo proliferative responses, ATC exhibited cytotoxic function after arming with EGFRBi.

Although our preclinical studies showed that EGFR BATs maintain their cytotoxicity after radiation and TMZ, it is possible that TMZ blunted the anti-tumor effect of immunotherapy [11]. Because the benefit of TMZ in patients with unmethylated MGMT GBM is controversial and its withholding for this group has been proposed [27, 28], we expanded the study to include three patients with unmethylated MGMT who received weekly EGFR BATs without adjuvant TMZ. The results of this cohort provided information for the design of a phase II study to include patients with unmethylated MGMT GBM.

Multiple infusions of EGFR BATs significantly increased GBM-specific CTL and NK activity, and serum concentrations of Th_1_ cytokines and chemokines. The evaluation of anti-tumor-specific cytotoxicity was limited to using tumor cell lines because we could not grow cell lines from the patient’s tumors. These findings are consistent with our earlier studies showing that PBMC isolated from patients after multiple infusions of BATs exhibit significant anti-tumor cytotoxicity and IFN-γ EliSpots responses to other types of cancer cell lines [14, 20, 22, 29, 30]. A potential advantage of our strategy is that the ability of EGFR BATs to recruit and activate endogenous immune cells in the TME may enhance systemic-specific cellular and humoral tumor immunity [18]. In combination, these data will inform clinical trials that examine the efficacy of this immunotherapy in patients with GBM.

Enrollment was limited by our exclusion of 20% of the patients with high IgE alpha-gal in serum that cross-react to cetuximab, a component of EGFRBi known to increase the risk of anaphylactic reactions [17, 31]. Before instituting the exclusion, an earlier trial using EGFR BATs in seven patients with unresectable and metastatic pancreatic cancer was safe without anaphylactic reactions [22]. In our clinical pipeline, a molecularly engineered recombinant anti-CD3 x anti-EGFR BiAb (rEGFRBi) with deletion of the sequence responsible for allergic reactions, has markedly enhanced cytotoxicity, and induced Th_1_ cytokine secretion when engaging multiple EGFR + tumor cell lines including GBM [32].

Although by IHC we detected EGFR in all nine samples examined (one sample was not available), there remains a concern that low or nil EGFR expression on GBMs would preclude targeting by EGFR BATs. Our preclinical studies showed that HER2 BATs could efficiently kill MCF-7 cells, a cell line with negative to low HER2 expression by flow cytometry [20]. In our phase I trial including patients with HER2-negative metastatic breast cancer (MBC) [21], we observed a near complete response in a patient with HER2-negative liver metastases and found a lack of correlation between HER2 status and survival. These results suggest that armed T cells may kill tumors with low or near nil TAA expression in the clinical setting.

Our study shows that EGFR BATs were produced from PBMC of patients with AG4 before receiving concomitant RT/TMZ, but that in some instances, T cell expansion was limited by tumor-induced immunosuppression or other inherent T cell dysfunction. We demonstrated that up to eight infusions of 10 × 10^9^ EGFR BATs were feasible, safe, and well tolerated in both every 4-week and weekly regimens while inducing cellular and cytokine/chemokine anti-glioma immune responses. These results support further studies in patients with GBM to demonstrate the efficacy of the safe and feasible dose of 80 × 10^9^ EGFR BATs. Future trials will involve an optimized rEGFRBi with the potential of enhanced T cell-mediated antitumor cytotoxicity [32], labeling of T cells to track trafficking into the GBM microenvironment, and combining EGFR BATs with other immune stimulatory agents.

### Supplementary Information

Below is the link to the electronic supplementary material.

Supplementary file1 (DOCX 19 KB)

Supplementary file2 (DOCX 13478 KB)

## Data Availability

To obtain the protocol and data from this study, please send a request to the corresponding author. The author will review the request and seek approval from the study investigators before providing access to the information.

## Abbreviations

AG4: Astrocytoma grade 4
ATC: Activated T-cells
BBB: Blood brain barrier
CNS: Central nervous system
CSF: Cerebrospinal fluid
CTL: Cytotoxic T lymphocytes
CR: Complete response or remission
DLT: Dose-limiting toxicity
EGFR: Epidermal growth factor receptor
EGFRBi: Anti-CD3 x anti-EGFR bispecific antibody
EGFR BATs: EGFRBi-armed activated T cells
GBM: Glioblastoma
GMP: Good Manufacturing Practice
HAHA: Human anti-human antibodies
HAMA: Human anti-mouse antibodies
HER2Bi: Anti-CD3 x anti-Her2 bispecific antibody
HER2 BATs: HER2Bi aATC
IHC: Immunohistochemistry
IFN-γ: Interferon gamma
IL-2: Interleukin-2
IT: Immunotherapy
IV: Intravenous
KPS: Karnofsky performance scale
mAb: Monoclonal antibody
MBC: Metastatic breast cancer
MGMT: O6-methylguanine-DNA methyltransferase
MHC: Major histocompatibility complex
MTFD: Maximum tolerated and feasible dose
ORR: Overall response rate
OS: Overall survival
PBMC: Peripheral blood mononuclear cells
PD: Progressive disease
PFS: Progression-free survival
PR: Partial response
RT: Radiation therapy
SC: Specific cytotoxicity
SD: Stable disease
TAA: Tumor-associated antigen
TILs: Tumor-infiltrating lymphocytes
TMZ: Temozolomide
