# Supplementary material for: Phase I study targeting newly diagnosed grade 4 astrocytoma with bispecific antibody armed T cells (EGFR BATs) in combination with radiation and temozolomide
Source: J Neurooncol. 2024 Jan 23;166(2):321–30. doi: 10.1007/s11060-024-04564-y (PMC10834565; doi:10.1007/s11060-024-04564-y)
Supplement: Supplementary file 1 — Supplementary file1 (DOCX 19 KB) [file 11060_2024_4564_MOESM1_ESM.docx]

**Phase I Study Targeting Newly Diagnosed Grade 4 Astrocytoma with Bispecific Antibody Armed T Cells (EGFR BATs) in Combination with Radiation and Temozolomide**

Camilo E. Fadul^1*^, Archana Thakur^2*^, Jungeun Kim^3^, Jessica Kassay-McAllister^2^, Dana Schalk^2^, M. Beatriz Lopes^4^, Joseph Donahue^5^, Benjamin Purow^1^, Patrick Dillon^6^, Tri Le^6^, David Schiff^1^, Qin Liu^7^ and Lawrence G. Lum^2^

**S**

**upplementary Methods**

**Phenotyping of Cell Therapy Product**

Cell therapy product ATC was stained with antibodies directed at CD3^+^, CD4^+^, CD8^+^, CD25^+^, CD19^+^, CD20^+^, CD45RO^+^, CD45RA^+^, CD16^+^, CD56^+^, CD127^+^, CD11b^+^, CD33^+^, and HLA-DR^+^ and analyzed by flow cytometry for cytotoxic T lymphocytes, T helper cells, T effector memory cells, NK cells, B cells, T regulatory cells (Tregs), and myeloid-derived suppressor cells (MDSC).

**^51^Chromium (^51^Cr) Release Assay for EGFR Specific Cytotoxicity by Product**

We used the U87 cell line, which expresses high levels of EGFR, as a target for the cytotoxicity assays. The target cell lines were plated in 96-well flat-bottom microtiter plates at 4 x 10^4^ cells/well, allowed to adhere overnight at 37^o^C, and labeled with ^51^Cr at 20 (µCi/mL) in the labeling media (50% fetal bovine serum (FBS) in complete RPMI-1640 medium supplemented with 10% FBS, 2% penicillin-streptomycin, and 1% L-glutamine as described (21). Effectors, unarmed or armed ATC (EGFR BATs) were then added to achieve an effector: target (E: T) ratio of 25:1. Co-cultures were incubated for 18 hours and the supernatants were collected for liquid scintillation counting to quantitate the amount of ^51^Cr released. Percent-specific cytotoxicity was calculated as follows: (experimental counts per minute (cpm) – spontaneous cpm) / (maximum cpm – spontaneous cpm) × 100. Maximum cytotoxicity was determined by adding 2% SDS. Means and standard errors were calculated from four to six replicates per sample.

**Immunohistochemistry Assay**

Immunohistochemistry (IHC) for EGFR (Thermo Scientific, 1:800) was performed using the automated protocol on a Ventana Discovery Ultra instrument (Roche Diagnostics, Ventana **Medical** Systems, Tucson, AZ). Sections of human tonsils known to contain abundant various immune cell populations were used as positive controls. Slides were scanned on the Hamamatsu slide scanner NanoZoomer S360 and images were viewed on the NDP viewer software. Analysis of images was performed on online Visiopharm software, this software is run on a server supported by the UVA ITS.  One of the investigators (MBL), who is a board-certified neuropathologist, marked the regions of interest within the tumor. The UVA Biorepository & Tissue Research Facility did the staining, scanning, and image analysis.

**Scoring of Cell Densities and Expression Intensities of Immunostained Sections.** IHC-stained slides were quantitatively analyzed in nine tumor biopsy sections. In one case, there was not enough tissue to do the IHC. EGFR expression cells were evaluated for overall impression at low microscopic magnification (40x) followed by higher magnification (100x– 200x) fields for tumor margin, stroma, and tumor center. The stained sections were scored as 0 (0-49/mm^2^) for the very low numbers, 1 (50-100 specific immune cells/mm^2^ with an increment of 0.1 with 10 cells) for sparse, 2 (101-1000 specific cells/mm^2^) for moderately dense, 3 (1001-5000 specific cells/mm^2^), 4 (5000-10,000 specific cells/mm^2^) for dense, and 5 (>10,000 specific immune cells/mm^2^) for very dense.

***Immune Monitoring Studies***

**Specific IFN-γ EliSpots**

All immune monitoring studies were performed on frozen cells or serum in a batch run to avoid sample-to-sample variation each time. Cytotoxicity was assessed in fresh PBMC from patients and normal controls against GBM cell line (U87) and NK cell target (K562) as targets for IFN-γ EliSpots to measure CD8 and CD4 mediated memory CTL and helper responses, respectively. IFN-γ EliSpots were assessed after 18 hours of stimulation with U87 or K562 at an effector-to-target ratio (E/T) of 1:1 as described[1]. Specific anti-GBM responses were corrected for spontaneous background IFN-γ EliSpots produced by PBMC.

**Serum cytokines**

Serum cytokine/chemokine profiles were measured at selected time points using a 25-plex human cytokine Luminex Array (R&D Systems, Minneapolis, MN) using the BioPlex system (Bio-Rad Lab., Hercules, CA). A 27-plex human cytokine/chemokine panel included IP-10, MIP-3β, MIP-1α, MIP-1β, RANTES, Fractalkine, IL-8, MCP-1, GRO-α, GRO-β, GM-CSF, IFN-γ, TNF-α, IL-2, IL-12, IL-7, L-15, IL-4, IL-5, IL-6, IL-10, IL-13, IL-33, GrzB, PD1, CD40L, Flt-3L.

**Reference**

1. Thakur A, Rathore R, Kondadasula SV, Uberti JP, Ratanatharathorn V, Lum LG (2018) Immune T cells can transfer and boost anti-breast cancer immunity. Oncoimmunology 7: e1500672 doi:10.1080/2162402X.2018.1500672
