# Supplementary material for: Phase I study targeting newly diagnosed grade 4 astrocytoma with bispecific antibody armed T cells (EGFR BATs) in combination with radiation and temozolomide
Source: J Neurooncol. 2024 Jan 23;166(2):321–30. doi: 10.1007/s11060-024-04564-y (PMC10834565; doi:10.1007/s11060-024-04564-y)
Supplement: Supplementary file 2 — Supplementary file2 (DOCX 13478 KB) [file 11060_2024_4564_MOESM2_ESM.docx]

**Phase I Study Targeting Newly Diagnosed Grade 4 Astrocytoma with Bispecific Antibody Armed T Cells (EGFR BATs) in Combination with Radiation and Temozolomide**

Camilo E. Fadul^1*^, Archana Thakur^2*^, Jungeun Kim^3^, Jessica Kassay-McAllister^2^, Dana Schalk^2^, M. Beatriz Lopes^4^, Joseph Donahue^5^, Benjamin Purow^1^, Patrick Dillon^6^, Tri Le^6^, David Schiff^1^, Qin Liu^7^ and Lawrence G. Lum^2^

**Table S1. Adverse events associated with 68 infusions of EGFR BATs (<10% of infusions)**

| **Adverse Event** | **Grade 1-2** | **Grade 3** | **Grade 4** | **Total No. of events (%)** |
| --- | --- | --- | --- | --- |
| Vomiting | 6 | 0 | 0 | 6 (9) |
| Leukopenia | 6 | 0 | 0 | 6 (9) |
| Confusion | 3 | 1 | 0 | 4 (6) |
| Back pain | 4 | 0 | 0 | 4 (6) |
| Hypokalemia | 1 | 3 | 0 | 4 (6) |
| Amnesia | 4 | 0 | 0 | 4 (6) |
| Anemia | 4 | 0 | 0 | 4 (6) |
| Respiratory, thoracic, and mediastinal disorders | 3 | 0 | 0 | 3 (4) |
| Skin and subcutaneous tissue disorders | 3 | 0 | 0 | 3 (4) |
| Abdominal pain | 3 | 0 | 0 | 3 (4) |
| Anorexia | 3 | 0 | 0 | 3 (4) |
| Seizure | 3 | 0 | 0 | 3 (4) |
| Nervous system disorders - Other, specify | 3 | 0 | 0 | 3 (4) |
| Flu-like symptoms | 3 | 0 | 0 | 3 (4) |
| Sinus tachycardia | 3 | 0 | 0 | 3 (4) |
| Tremor | 3 | 0 | 0 | 3 (4) |
| Dysphasia | 2 | 0 | 0 | 2 (3) |
| Gait disturbance | 2 | 0 | 0 | 2 (3) |
| Insomnia | 2 | 0 | 0 | 2 (3) |
| Muscle weakness lower limb | 2 | 0 | 0 | 2 (3) |
| Diarrhea | 2 | 0 | 0 | 2 (3) |
| Eye disorders - Other, specify | 2 | 0 | 0 | 2 (3) |
| Sore throat | 2 | 0 | 0 | 2 (3) |
| Lethargy | 1 | 0 | 0 | 1 (2) |
| Dysgeusia | 1 | 0 | 0 | 1 (2) |
| Muscle weakness left-sided | 1 | 0 | 0 | 1 (2) |
| Hypertension | 1 | 0 | 0 | 1 (2) |
| Movements involuntary | 1 | 0 | 0 | 1 (2) |
| Palpitations | 1 | 0 | 0 | 1 (2) |
| Peripheral sensory neuropathy | 1 | 0 | 0 | 1 (2) |
| Sinus bradycardia | 1 | 0 | 0 | 1 (2) |
| Musculoskeletal | 1 | 0 | 0 | 1 (2) |
| Memory impairment | 1 | 0 | 0 | 1 (2) |
| Nervous system disorders - Other | 1 | 0 | 0 | 1 (2) |
| Myalgia | 1 | 0 | 0 | 1 (2) |
| Bruising | 1 | 0 | 0 | 1 (2) |
| Sinus bradycardia | 1 | 0 | 0 | 1 (2) |
| Tinnitus | 1 | 0 | 0 | 1 (2) |
| Nasal congestion | 1 | 0 | 0 | 1 (2) |
| Intracranial hemorrhage | 1 | 0 | 0 | 1 (2) |
| Constipation | 1 | 0 | 0 | 1 (2) |
| Anxiety | 1 | 0 | 0 | 1 (2) |
| Neutropenia | 1 | 0 | 0 | 1 (2) |
| Dizziness | 1 | 0 | 0 | 1 (2) |
| Depression | 1 | 0 | 0 | 1 (2) |
| Cognitive disturbance | 1 | 0 | 0 | 1 (2) |
| Fall | 1 | 0 | 0 | 1 (2) |

**Table S2A. Summary of product characteristics**

| **Patient** | **Total Harvest x 10^9^** | **Viability**  **%** | **%CD3 Post Harvest** | **%CD4 Post Harvest** | **%CD8 Post Harvest** | **CD4/CD8 ratio** | **% Cytox (E/T Ratio 25:1)** |
| --- | --- | --- | --- | --- | --- | --- | --- |
|  |  |  |  |  |  |  |  |
| GBM6 | **73.9** | **78.5** | 93.74 | 54.07 | 50.63 | 1.07 | **30.1** |
| GBM7 | **72.7** | **89.6** | 94.92 | 39.1 | 59.12 | 0.66 | **20.9** |
| GBM9 | **84.5** | **71.1** | 92.75 | 31.53 | 62.12 | 0.51 | **42.4** |
| GBM11 | **68.1** | **92.4** | 99.05 | 40.8 | 59.2 | 0.69 | **43.4** |
| GBM12 | **108** | **72.6** | 97.42 | 32.06 | 66.77 | 0.48 | **55.4** |
| GBM14 | **106.3** | **85.5** | 97.5 | 39.16 | 57.4 | 0.68 | **60** |
| GBM15 | **109.1** | **80.5** | 90.61 | 64.96 | 33.24 | 1.95 | **58.3** |
| GBM17 | **77** | **94.9** | 91.12 | 56.08 | 44.56 | 1.26 | **52.9** |
| GBM21 | **77.4** | **91.9** | 95.54 | 33.64 | 65.97 | 0.51 | **39.2** |
| GBM23 | **114** | **80.8** | 96.02 | 44.44 | 55.68 | 0.8 | **47.9** |
| **MEAN** | **84.2** | **83.0** | 94.9 | 43.6 | 55.5 | 0.9 | **43.4** |
| **RANGE** | **68.1 – 114** | **71.1 – 94.9** | **90.6 – 99.1** | **31.5 – 65.0** | **33.2 – 66.7** | **0.48-1.95** | **20.9 –60.07** |

**Cytox: cytotoxicity; E: effector; T: target**

**Table S2B. Summary of product immune cell phenotype**

**T_EM_ = Effector Memory T cells**

**T_EMRA_= T_EM_ re-expressing CD45RA**

**Tregs=T regulatory cells**

**MDSC=Myeloid derived suppressor cells**

| **Patient ID** | **NKT cells** | **NK cells** | **B cells** | **Naïve CD4 T cells** | **CD4 T_EM_ cells** | **CD4 T_EMRA_** | **Naïve CD8 T cells** | **CD8 T_EM_ cells** | **CD8 T_EMRA_** | **Tregs** | **MDSC** |
| --- | --- | --- | --- | --- | --- | --- | --- | --- | --- | --- | --- |
| **GBM6** | 0.4 | 1.56 | 0.6 | 2.17 | 52.12 | 0.73 | 1.91 | 30.75 | 2.17 | 4.21 | 0.07 |
| **GBM7** | 0.3 | 0.45 | 0.04 | 0.17 | 30.68 | 0 | 1.73 | 20.64 | 0.06 | 0.27 | 0 |
| **GBM9** | 9.26 | 0.65 | 0.34 | 0.66 | 28.85 | 0.29 | 9.31 | 66.6 | 1.99 | 0.02 | 0.2 |
| **GBM11** | 0.49 | 0.53 | 0.81 | 6.34 | 23.45 | 22.39 | 26.39 | 12.38 | 23.47 | 1.68 | 0.68 |
| **GBM12** | 1.97 | 2.05 | 0.48 | 0.16 | 40.93 | 11.31 | 0.47 | 63.7 | 22.67 | 1.16 | 0.02 |
| **GBM14** | 0.5 | 0.51 | 0.23 | 1.28 | 53.36 | 5.76 | 2.22 | 40.09 | 10.19 | 3.95 | 0.34 |
| **GBM15** | 3.2 | 3.85 | 0.81 | 0.35 | 50.68 | 13.88 | 0.86 | 15.58 | 11.31 | 0 | 0 |
| **GBM17** | 1.34 | 1.3 | 3.99 | 0.24 | 48.96 | 4.63 | 0.55 | 52.67 | 4.91 | 0.14 | 0.02 |
| **GBM21** | 0.96 | 1.37 | 0.94 | 10.68 | 20.68 | 9.32 | 24.66 | 11.3 | 24.71 | 2.33 | 0.98 |
| **GBM23** | 2.22 | 1.09 | 0.3 | 0.31 | 46.83 | 4.9 | 0.78 | 25.04 | 8.46 | 1.24 | 1.35 |
| **Mean** | **2.1** | **1.3** | **0.9** | **2.2** | **39.7** | **7.3** | **6.9** | **33.9** | **11.0** | **1.5** | **0.4** |
| **Range** | **0.3-9.26** | **0.45-3.85** | **0.04-3.99** | **0.17-10.68** | **20.68-53.36** | **0-22.9** | **0.47-26.39** | **11.3-66.6** | **0.06-24.71** | **0-4.21** | **0-1.35** |

**Table S3.** Fold changes in cytokines and chemokines PostIT compared to PreIT baseline

| **Patient** | **IFN-γ** | **IL-2** | **GM-CSF** | **IL-10** | **MIP-1β** | **IP-10** | **CX3CL1** | **RANTES** | **CD40L** | **Flt-3L** |
| --- | --- | --- | --- | --- | --- | --- | --- | --- | --- | --- |
| **GBM6** | **5.7** | **9.4** | **24.8** | **2.5** | **20.3** | **2.7** | **2.3** | **0.9** | **1.3** | **1.2** |
| **GBM7** | **1.2** | **2.3** | **2.4** | **29** | **8.1** | **2.3** | **1.5** | **1.5** | **1.1** | **2.8** |
| **GBM9** | **1.2** | **0.9** | **0.8** | **1.5** | **1.2** | **1.6** | **1.1** | **1** | **1.2** | **1.2** |
| **GBM11** | **1** | **2.8** | **2.1** | **2** | **1.1** | **1.8** | **1.5** | **2.9** | **1.1** | **3** |
| **GBM12** | **1.4** | **2.5** | **0.9** | **1.1** | **1** | **0.9** | **1.3** | **1.7** | **4.3** | **1.8** |
| **GBM14** | **1** | **1** | **1.6** | **1.8** | **1.3** | **1.8** | **1.6** | **5.3** | **1.3** | **1.9** |
| **GBM15** | **1** | **5.1** | **1.5** | **1.4** | **1.5** | **1.5** | **1.8** | **3.9** | **2.5** | **4.8** |
| **GBM17** | **1** | **2.1** | **1.2** | **0.9** | **1.1** | **0.8** | **1** | **0.6** | **0.9** | **2.8** |
| **GBM21** | **45.2** | **26.7** | **1.7** | **1.9** | **1.5** | **1.1** | **1.9** | **2.7** | **9.8** | **3.5** |
| **GBM23** | **68.7** | **5.6** | **6.6** | **4.4** | **2.6** | **2.3** | **2.6** | **0.8** | **2.5** | **2** |

**Supplementary Figures**

**Figure S1**


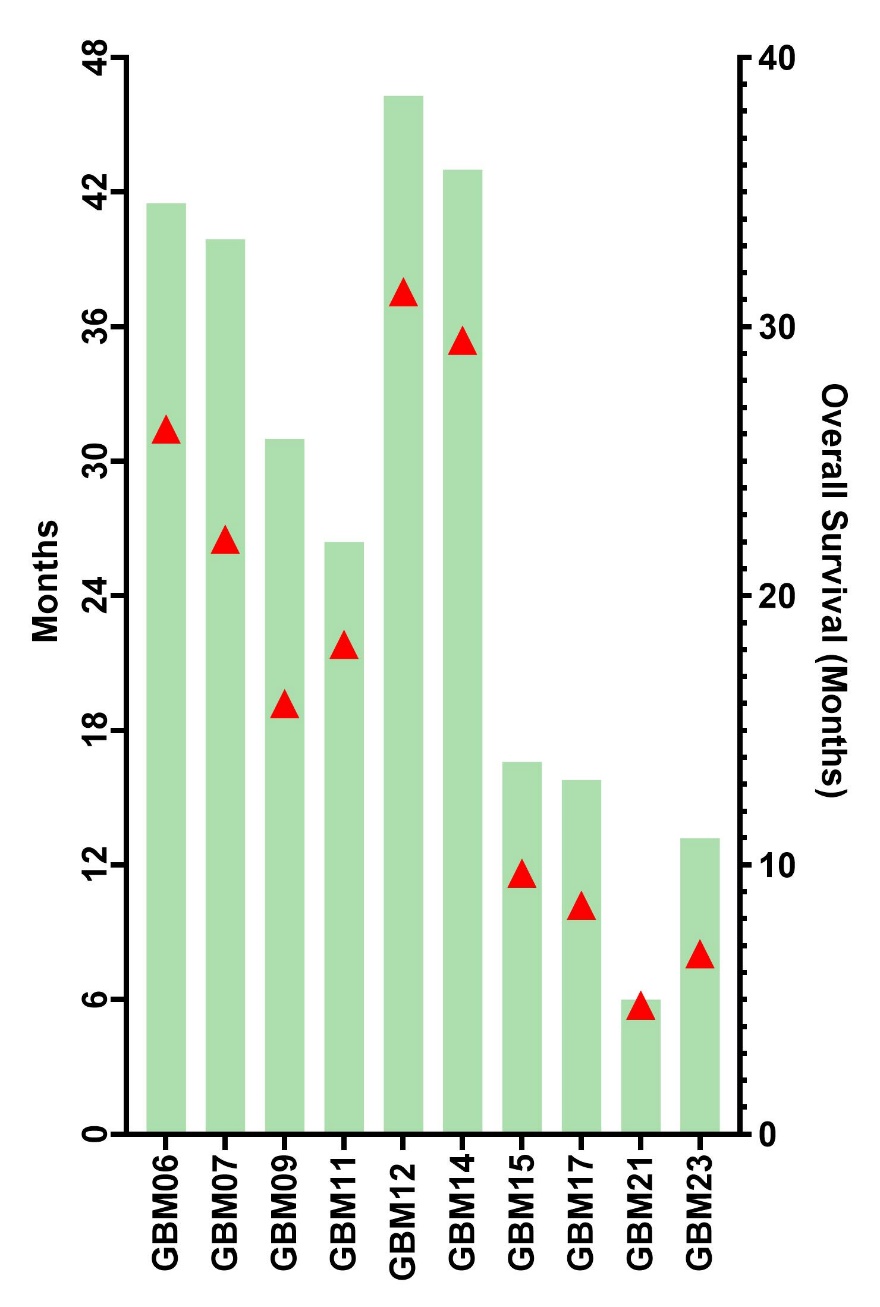

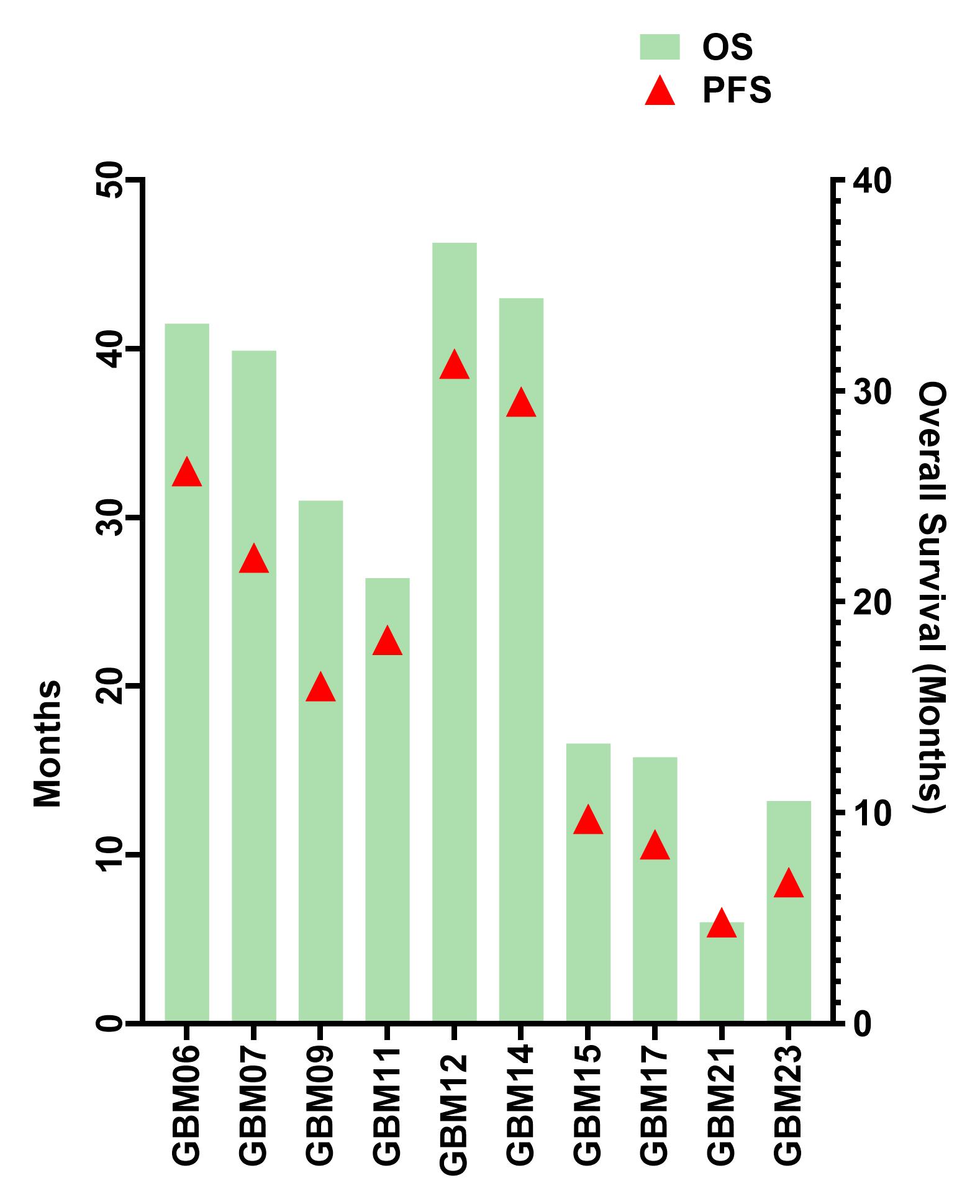


**Disease Progression**

**Survival Status**

**Figure S1.** The swimmer plot shows the progression-free survival and overall survival for each patient.

**Figure S2**

**
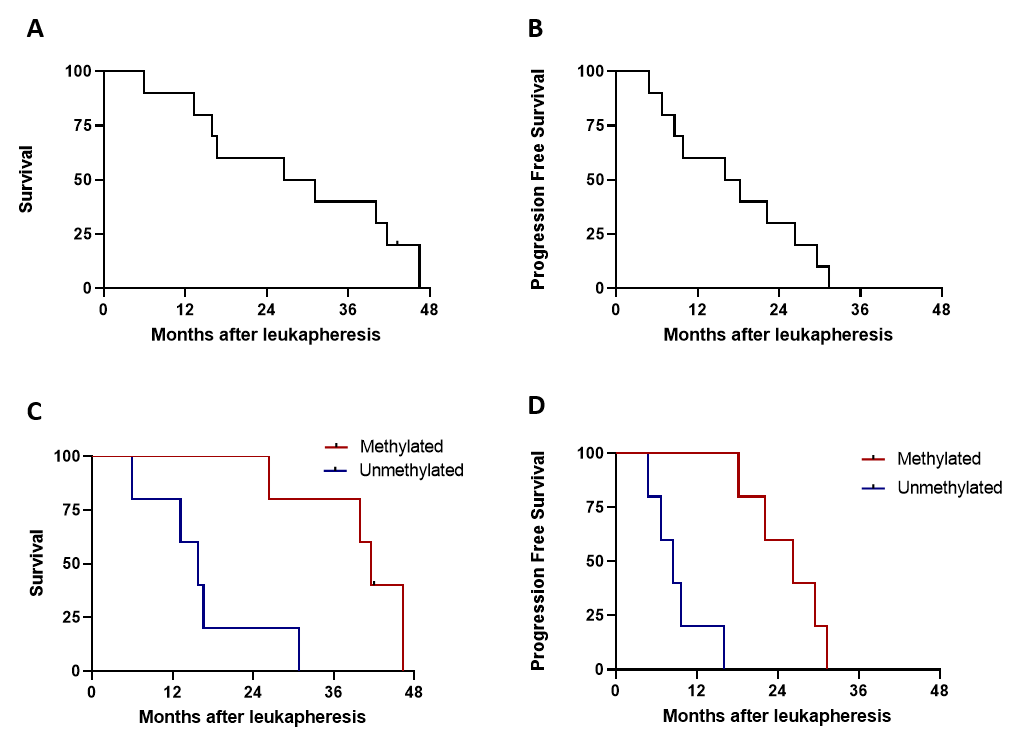
**

**Figure S2.** Kaplan-Meier curves show the survival (A) and progression-free survival (B) for all patients and the survival (C) and progression-free survival (D) according to MGMT gene promoter methylation.

**
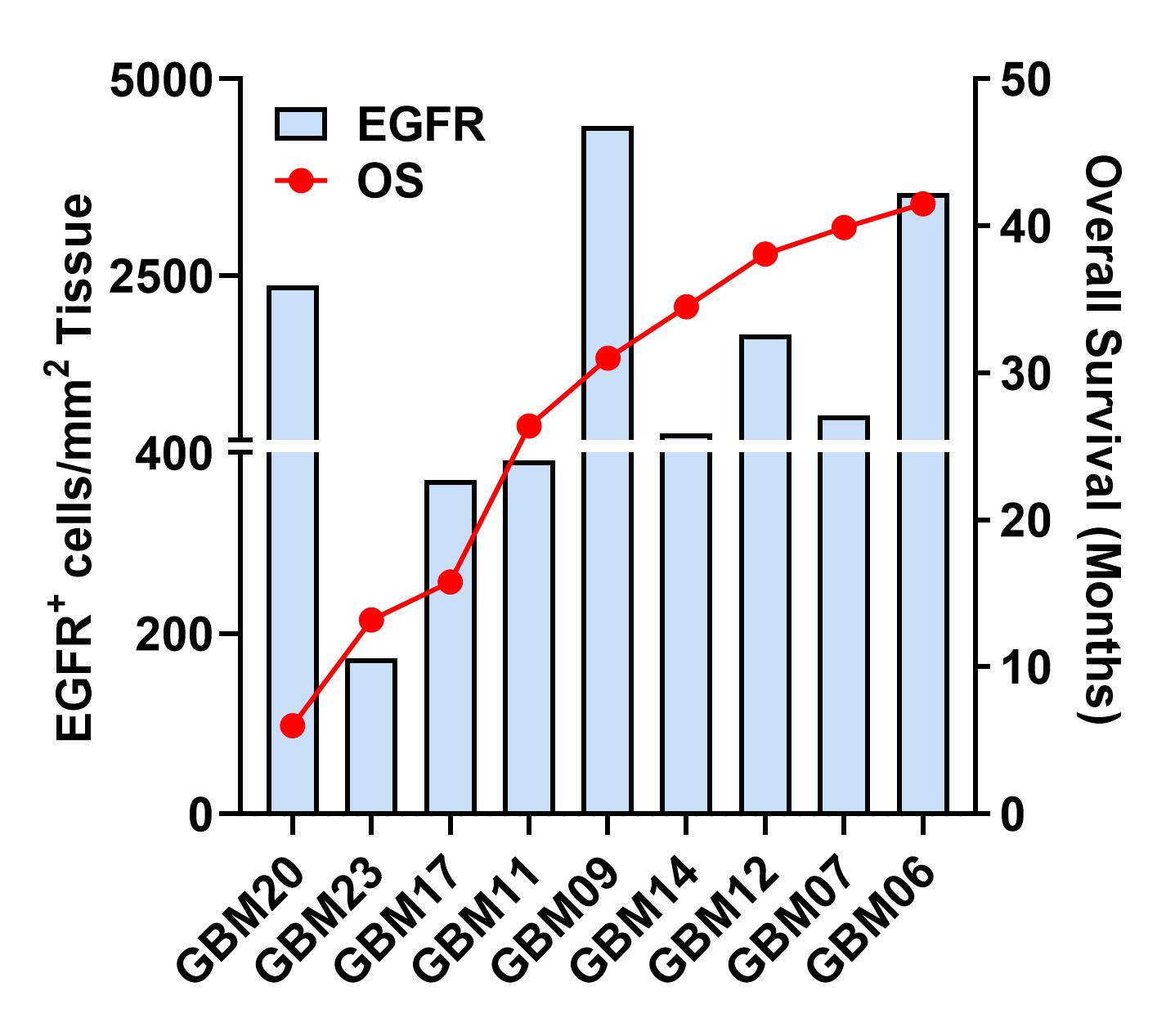
Figure S3**

**Figure S3.** Quantitation data for immunohistochemistry for EGFR as counts in a defined tumor area on the left *y*-axis and OS on the right *y*-axis for each patient.

**Figure S4**

**EGFR Expression**

**GBM-11**

**GBM-23**

**GBM-12**

**GBM-07**

**GBM-20**

**GBM-06**

**GBM-17**

**GBM-14**

**GBM-09**


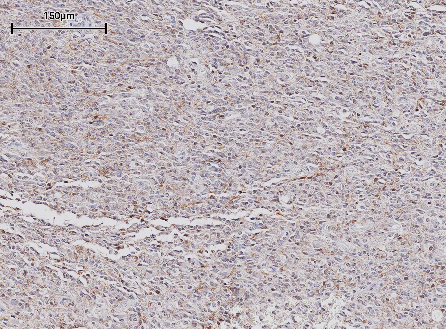

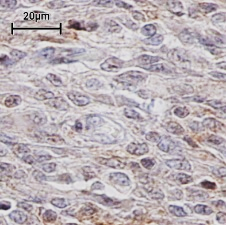

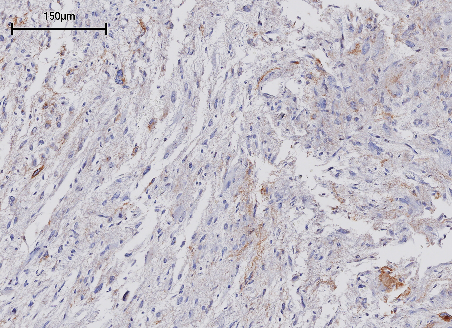

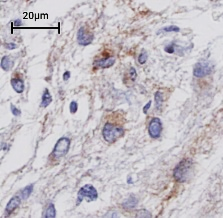

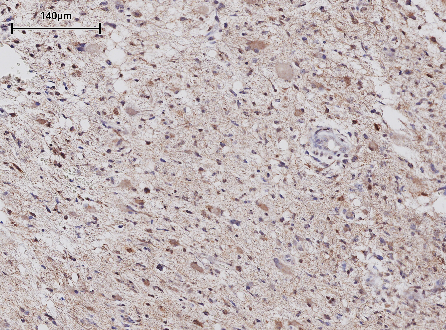

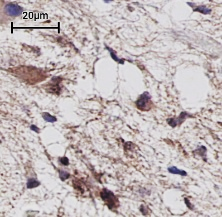

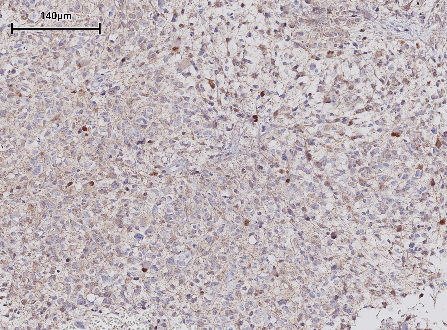

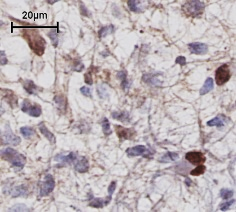

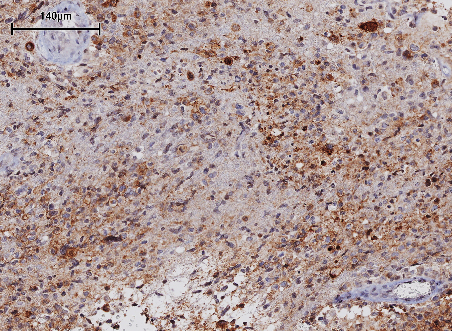

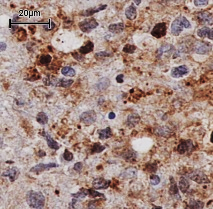

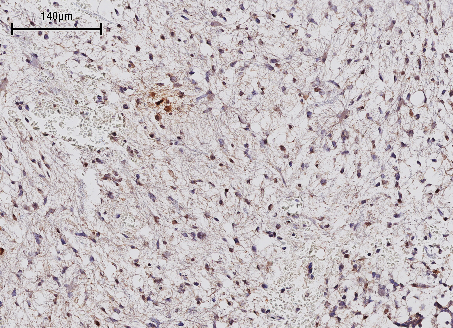

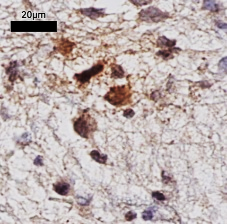

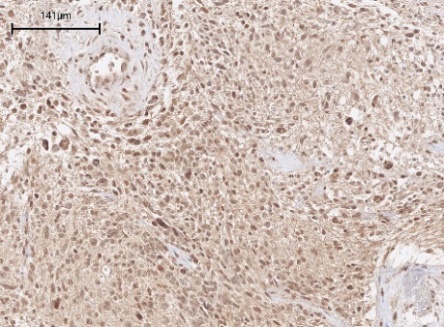

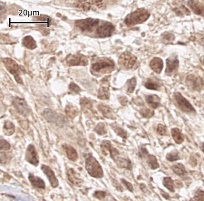

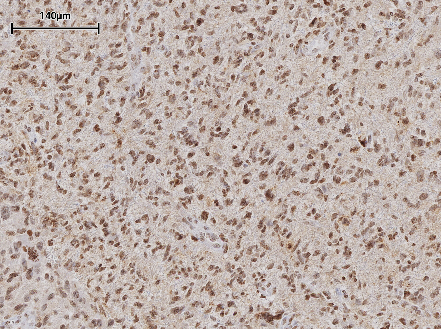

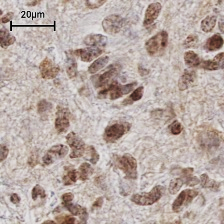

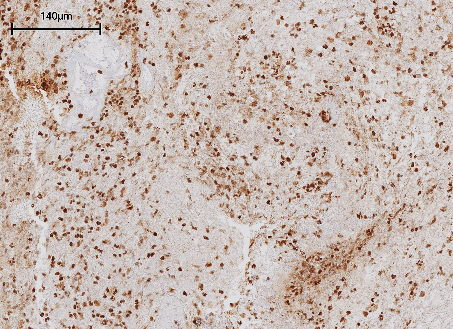

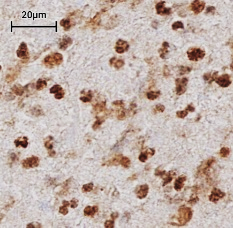


**Figure S4.** Shows IHC images of EGFR labeling in nine of 10 patients’ diagnostic biopsies, the inset shows the positive cells in 20μm tumor tissue area.
